# P-662. Impact of Antibiotic Exposures at Intubation on Development of Ventilator-Associated Pneumonia in Trauma Patients

**DOI:** 10.1093/ofid/ofaf695.875

**Published:** 2026-01-11

**Authors:** Michelle Fang, Tinh Duong, Allison Osvog, Jeff Jolliff

**Affiliations:** Kern Medical, Bakersfield, CA; Kern Medical, Bakersfield, CA; Kern Medical, Bakersfield, CA; Kern Medical, Bakersfield, CA

## Abstract

**Background:**

Recent literature supports the use of single-dose ceftriaxone within 12 hours of endotracheal intubation to prevent ventilator-associated pneumonia (VAP) in patients with acute brain injury. As patients admitted for trauma may receive antibiotics around the time of intubation for other prophylactic indications, we sought to assess the association between antibiotic exposures and the development of VAP in trauma patients at Kern Medical (KM), a level 2 trauma center in Bakersfield, CA.

**Methods:**

We performed a case-control study of patients admitted to the trauma service at KM who required mechanical ventilation (MV) from January 1, 2023 to June 30, 2024. Patients who developed VAP, as defined by the American College of Surgeons Trauma Quality Improvement Program, were included as cases; patients without VAP were included as controls. Exclusion criteria were MV for less than 48 hours or admission to KM for less than 72 hours. Demographic and outcomes data were extracted from the trauma registry while antibiotic use data was obtained through chart review. Antibiotic exposures and clinical outcomes were compared between cases and controls.

**Results:**

Of the 84 intubated trauma patients identified over the study period, 18 cases and 48 controls were included. Characteristics at admission included average age 40 years (VAP 44 years vs no VAP 37 years), injury severity score 24 (VAP 27 vs no VAP 24), probability of survival 0.81 (VAP 0.81 vs no VAP 0.81), and traumatic brain injury (TBI) 52% (VAP 44% vs no VAP 54%). Average time from intubation to development of VAP was 5 days. Those with VAP were less likely to have received ceftriaxone or antibiotics with a similar spectrum of activity within 24 hours of intubation (OR 0.09, 95% CI 0.02-0.43). All-cause mortality was 21% (VAP 11% vs no VAP 25%). At 28 days post-intubation, cases had an average of 11 ventilator-free days and 14 antibiotic-free days while controls had 17 ventilator-free days and 14 antibiotic-free days.

**Conclusion:**

Receipt of ceftriaxone or equivalent antibiotic within 24 hours of intubation appeared to be a negative risk factor for VAP. Increased mortality among controls may have been attributable to higher TBI incidence. Interestingly, although controls had more ventilator-free days, antibiotic-free days was similar between groups.

**Disclosures:**

All Authors: No reported disclosures

